# Response rate of anticancer drugs approved by the Food and Drug Administration based on a single-arm trial

**DOI:** 10.1186/s12885-022-09383-w

**Published:** 2022-03-15

**Authors:** Yoshihiro Oda, Mamoru Narukawa

**Affiliations:** 1 Department of Clinical Medicine (Pharmaceutical Medicine), Graduate School of Pharmaceutical Sciences, Kitasato University, Shirokane 5-9-1, Minato-ku, Tokyo, 108-8641 Japan; 2 Chugai Pharmaceutical Co, Ltd, Nihonbashi-Muromachi 2-1-1, Chuo-ku, Tokyo, 103-8324 Japan

**Keywords:** Anticancer drug, Pivotal trial, Response rate, Single-arm trial

## Abstract

**Background:**

In recent years, an increasing number of anticancer drugs have been approved based on the results of a single-arm trial (SAT). The magnitude of the objective response rate (ORR) in SATs is important for regulatory decisions, but there has been no clear guidance specifying the degree of ORR for approval.

**Methods:**

All anticancer drugs approved by the US Food and Drug Administration (FDA) between January 2016 and December 2019 were identified through the FDA website. From these, we selected drugs approved for solid tumors based on SATs. For each indication, one regimen was selected from the standard-of-care as the best comparison therapy (BCT), which was defined as the latest regimen for the same tumor and treatment line. We compared the ORR of the investigated product with that of the BCT.

**Results:**

Of the 31 solid tumor indications identified, we selected BCT for 28. In 23 of the 28 indications (82.1%), the ORR of the investigated product exceeded that of the BCT, and in 16 of these (69.6%), the lower limit of the 95% confidence interval (CI) of the ORR of the investigated product exceeded the point estimate of the BCT ORR. For seven products, the lower limit of the 95% CI was below the point estimate of the BCT ORR, with differences ranging from 1.0% to 3.4%.

**Conclusion:**

The lower limit of a 95% CI of the ORR of a new drug in an SAT exceeding the point estimate of the BCT ORR could be an important factor in obtaining regulatory approval.

## Background

Development of an anticancer drug from inception through efficacy and safety evaluation is a stepwise process [1]. The maximum tolerated dose is explored in phase I studies, and the efficacy and safety of the dosage and administration thus determined are investigated in a targeted patient population in phase II studies. Subsequently, phase III studies are conducted to compare the efficacy and safety of the new drug against a standard treatment.

Since the 1980s, new anticancer drugs have been approved based on direct clinical benefits, such as prolonged survival and improved quality of life [2]. Typically, obtaining regulatory approval for new anticancer drugs involved demonstrating favorable results in randomized controlled trials (RCTs) with a primary endpoint, such as overall survival (OS). Approval was sometimes granted based on the results of a phase II study with a single-arm trial (SAT) design (without control arms), due to the difficulty in conducting RCTs for cancers with a small number of patients or for rare fractions with infrequent genetic abnormalities. Recently, anticancer drugs have increasingly been approved based on an SAT [3]. Advances in medicine and technology that have led to the development of effective drugs and genomic diagnostics for rare cancers and fractions underlie this trend. Thus, the number of SAT-based approvals is expected to increase.

Different filing strategies can be adopted for each drug; some require confirmatory phase III studies for filing, and some are accepted for filing with an earlier exploratory phase II study. In either case, a pivotal trial must show clinical benefits in the targeted patient population. The true endpoint for anticancer drugs is OS. To confirm this clinical benefit, RCTs should be conducted with a sample size that is calculated by setting statistically appropriate power and significance levels, so that superiority or non-inferiority of the new drug over the control arm can be tested. Moreover, subjects should be randomized by considering important prognostic factors.

In contrast, the primary endpoint used in SATs is the objective response rate (ORR). To demonstrate the clinical significance of the ORR, the expected response rate of the new drug must exceed the threshold response rate, based on the response rate to a standard-of-care. The Response Evaluation Criteria in Solid Tumors (RECIST) guideline version 1.1 [4] is commonly used for evaluation of ORRs. Evaluation involves measuring the tumor diameter based on computed tomography (CT) and/or other images, with evaluator-dependent results. Thus, evaluation by investigators may be biased, and hence ORRs evaluated by blinded independent central review are often used as a primary endpoint. Regulatory review, based on data from SATs, has to be conducted with limited information, because the ORR does not necessarily correlate with OS, depending on the cancer type. However, the ORR has advantages for the development of new drugs for rare cancers, where evaluation of the OS benefit compared to a standard-of-care is difficult. This approach can reduce development costs, shorten development time, and accelerate patient access to new drugs.

The guidance document on expedited programs for serious conditions by the US Food and Drug Administration (FDA) [5] states that “radiographic evidence of tumor shrinkage (response rate) in certain cancer types has been considered reasonably likely to predict an improvement in overall survival” as an example of an endpoint for approval by the accelerated approval (AA) scheme. Another guideline [2] states that “the FDA has sometimes accepted ORR and the response duration observed in single-arm studies as substantial evidence supporting accelerated approval.” Consequently, the magnitude of the ORR is important, and in general, decisions are made based on a high ORR [6]. However, because the magnitude of a clinically meaningful ORR expected for a new drug differs depending on the cancer type and line of treatment, the magnitude of an ORR required for approval differs depending on each indication. There are currently no clear guidelines specifying the degree of the ORR for regulatory approval, and reviews are conducted for individual drug situations. Additionally, no study has investigated the difference in the ORRs of an approved drug and a historical control.

This study explored the magnitude of the ORR necessary for granting regulatory approval by comparing the ORR of an anticancer drug approved by the FDA, based on SATs, with that of the standard-of-care that was considered as a historical control for the drug.

## Materials and methods

### Identification of products to be investigated and acquisition of relevant information

All anticancer drugs, including those for additional indications, approved by the FDA between January 2016 and December 2019, were identified through the FDA's Hematology/Oncology (Cancer) Approvals & Safety Notifications website [7], as of January 2020. If multiple indications were approved for a single product on the same day, each indication was counted separately. We excluded approvals for cellular and gene therapies, approvals with no anticancer effect indications, and those related to hematological malignancies, to extract approvals for indications for solid tumors. Next, we selected SAT-based (without control arms) approvals, by referring to the design of the pivotal trial on which approval was based. Among these, approvals for tumor agnostic indications and indications for which the ORR was not the primary endpoint were excluded, as we could not compare the ORR of the product with that of the standard-of-care.

We obtained data on the ORR and 95% confidence interval (CI) in the pivotal SAT from the product label. We also collected information on the indication and the mechanism of action (MOA) of the product from the label and on the application of special programs, such as breakthrough therapy designation, AA, fast track, priority review, and orphan drug designation, from the approval announcement for the product on the FDA website [7].

### Selection of the BCT and acquisition of relevant information

For each of the investigated products and approved indications, best comparison therapy (BCT) information was referenced to the most recent National Comprehensive Cancer Network clinical practice guidelines in oncology (NCCN guidelines) at the time of its approval. For original new drug applications for which the review report was available on the FDA website [8], we also referred to the treatment options listed in Chapter 2.2, “Analysis of current treatment options,” of the review report. For products and approved indications for which publications of the pivotal trial results were available, treatments listed as comparators in the introduction or discussion sections of the published articles were also referenced.

For each of the investigated products, we first identified the standard-of-care for the target tumor and treatment line. In cases where the patient population was limited by biomarkers and where there was no similar drug for populations with the same biomarkers, the drug was considered as first-in-class, and the standard-of-care used for patients not stratified by the biomarkers was considered to be a BCT. Second, in cases where there were multiple competing standard-of-care regimens, the most current regimen at the time of approval was selected as a BCT.

### Analysis

A scatter plot was created by comparing the ORR of the investigated product (with its 95% CI) with that of the BCT. No statistical analyses or tests were performed.

## Results

### Identification of investigated products

We identified 155 anticancer drug approvals between January 2016 and December 2019. We excluded three approvals for cellular therapy (two of tisagenlecleucel and one of axicabtagene ciloleucel), and four approvals related to indirect anticancer effects (subcutaneous use of a rituximab plus hyaluronidase combination for follicular lymphoma, diffuse large B-cell lymphoma, and chronic lymphocytic leukemia, subcutaneous use of trastuzumab plus hyaluronidase-oysk for breast cancer, lower-dose cabazitaxel for prostate cancer, and longer-acting calaspargase pegol-mknl for acute lymphoblastic leukemia). Forty-seven approvals for hematological malignancy were also excluded.

**Fig. 1:**
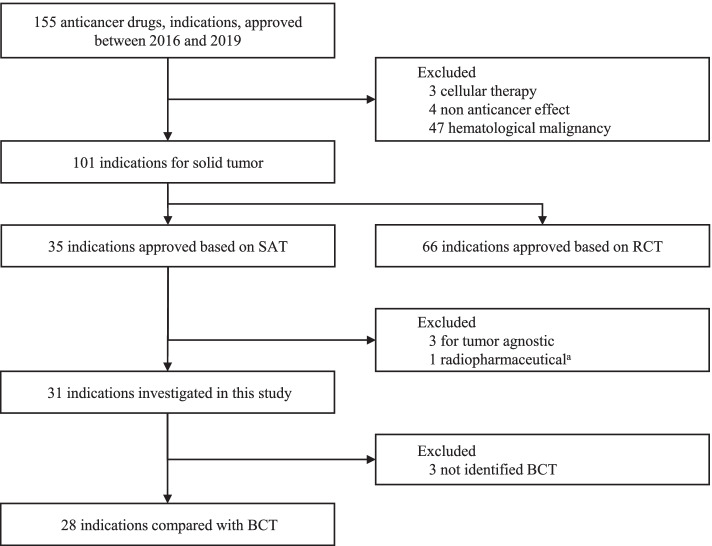
Identification of investigated products. *ORR* overall response rate, *RCT* randomized clinical trial, *SAT* single-arm trial, *BCT* best comparison therapy. ^a^ORR was not the primary endpoint in the pivotal SAT

Among 101 indications for solid tumors, approval was SAT-based for 35 and RCT-based for 66. From the 35 SAT approvals, three approvals of pembrolizumab, larotrectinib, and entrectinib for tumor agnostic indications were excluded, due to difficulty in comparing the results for each indication. One approval of iobenguane I^131^ was excluded because an endpoint other than the ORR was evaluated for approval. Consequently, 31 indications for solid tumors that were approved based on the SAT results were identified in this study (Fig. 1).

### Characteristics of approved indications for solid tumors

Table 1 shows the characteristics of approved indications for solid tumors: 35 were SAT- based and 66 were RCT-based. With regard to the cancer type for which the indication was approved, the cancer types with the highest number of indications approved based on RCTs were lung cancer (15 approvals [22.7%]) and breast cancer (14 [21.2%]), while the cancer types with the highest number of indications with SAT-based approval were lung cancer (8 [22.9%]) and bladder cancer (7 [20.0%]). For kidney cancer, prostate cancer, and neuroendocrine tumors, no drug was approved based on SAT results. On the other hand, all drugs for tissue/site agnostic indications and for colorectal cancer were approved based on SAT results.

**Table 1:** Characteristics of oncology drug approvals

		SAT n (%) *n* = 35	RCT n (%) *n* = 66
Approval Year	2016	4 (11.4)	9 (13.6)
	2017	12 (34.3)	16 (24.2)
	2018	11 (31.4)	22 (33.3)
	2019	8 (22.9)	19 (28.8)
Cancer Type	Bladder	7 (20.0)	1 (1.5)
	Breast	2 (5.7)	14 (21.2)
	Colorectal	2 (5.7)	0
	Gastric	1 (2.9)	1 (1.5)
	Head and Neck	1 (2.9)	2 (3.0)
	Kidney	0	7 (10.6)
	Liver	2 (5.7)	4 (6.1)
	Lung	8 (22.9)	15 (22.7)
	Neuroendocrine tumors	0	2 (3.0)
	Ovarian	2 (5.7)	5 (7.6)
	Prostate	0	6 (9.1)
	Skin	3 (8.6)	4 (6.1)
	Tumor agnostic	3 (8.6)	0
	Other	4 (11.4)	5 (7.6)
Mechanism of Action	Antibody drug conjugate	2 (5.7)	1 (1.5)
	Androgen receptor inhibitor	0	6 (9.1)
	Immune checkpoint inhibitor	18 (51.4)	19 (28.8)
	Molecularly-targeted drug	11 (31.4)	34 (51.5)
	Combo	3 (8.6)	3 (4.5)
	Other	1 (2.9)	3 (4.5)
Review Process	Breakthrough therapy	22 (62.9)	21 (31.8)
	Accelerated approval	26 (74.3)	3 (4.5)
	Fast track	2 (5.7)	5 (7.6)
	Priority review	34 (97.1)	46 (69.7)
	Orphan	10 (28.6)	14 (21.2)

*RCT* randomized clinical trial, *SAT* single-arm trial

With regard to the MOA of the drug, molecular targeted agents accounted for 51.5% (34/66) among the RCT-based approvals, while immune checkpoint inhibitors accounted for 51.4% (18/35) among the SAT-based approvals. No androgen receptor inhibitors were approved based on SAT results.

Among the 35 approved indications based on SATs, 22 (62.9%) had breakthrough therapy designation, 26 (74.3%) obtained AA, and 34 (97.1%) were subject to priority review.

### Identification of best comparison therapy

The treatments identified as BCTs for each of the 31 approved indications are shown in Table 2 [9–28]. For avelumab (#6) and pembrolizumab (#13), chemotherapy was used in clinical practice, but there is no standard or consensus regimen. For nivolumab (#20), best supportive care was used in clinical practice as the standard-of-care for this treatment line. For the other 28 indications, we could identify a BCT according to the criteria stated above (Fig. 1).

**Table 2:** List of investigated products

**#**	Product	FDA Approved Date	Indication	ORR	BCT	ORR of BCT	Reference of BCT
1	Crizotinib (Xalkori)	March 11, 2016	Metastatic NSCLC whose tumors are ROS1-positive	66.0%	Paclitaxel + Carboplatin + Bevacizumab	35%	Sandler et al.[9]
2	Atezolizumab (Tecentriq)	May 18, 2016	Locally advanced or metastatic UC who have disease progression during or following platinum-containing chemotherapy or have disease progression within 12 months of neoadjuvant or adjuvant treatment with platinum-containing chemotherapy	14.8%	Vinflunine	9%	Drugs@FDA [10]
3	Pembrolizumab (Keytruda)	August 5, 2016	Recurrent or metastatic head and neck squamous cell carcinoma with disease progression on or after platinum-containing chemotherapy	16.0%	Cetuximab	13%	Vermorken et al. [11]
4	Rucaparib (Rubraca)	December 19, 2016	Deleterious BRCA mutation (germline and/or somatic) associated advanced ovarian cancer who have been treated with two or more chemotherapies	54.0%	Olaparib	34%	Drugs@FDA [12]
5	Nivolumab (Opdivo)	February 2, 2017	Locally advanced or metastatic UC who have disease progression during or following platinum-containing chemotherapy or have disease progression within 12 months of neoadjuvant or adjuvant treatment with a platinum-containing chemotherapy	19.6%	Atezolizumab	14.8%	See the result of #2
6	Avelumab (Bavencio)	March 23, 2017	Metastatic MCC	33.0%	NA		
7	Brigatinib (Alunbrig)	April 28, 2017	Metastatic ALK-positive NSCLC who have progressed on or are intolerant to crizotinib	53.6%	Alectinib	44%	Drugs@FDA [13]
8	Durvalumab (Imfinzi)	May 1, 2017	Locally advanced or metastatic UC who have disease progression during or following platinum-containing chemotherapy or who have disease progression within 12 months of neoadjuvant or adjuvant treatment with platinum-containing chemotherapy	17.0%	Nivolumab	19.6%	See the result of #5
9	Avelumab (Bavencio)	May 9, 2017	Locally advanced or metastatic UC whose disease progressed during or following platinum-containing chemotherapy or within 12 months of neoadjuvant or adjuvant platinum-containing chemotherapy	16.1%	Nivolumab	19.6%	See the result of #5
10	Pembrolizumab (Keytruda)	May 18, 2017	Locally advanced or metastatic UC who are not eligible for cisplatin-containing chemotherapy	28.6%	Carboplatin + Gemcitabine	36.1%	Santis et al. [14]
11	Dabrafenib and Trametinib (Tafinlar and Mekinist)	June 22, 2017	Metastatic NSCLC with BRAF V600E mutation	61.0%	Paclitaxel + Carboplatin + Bevacizumab	35%	Sandler et al. [9]
12	Nivolumab (Opdivo)	July 31, 2017	dMMR and MSI-H metastatic colorectal cancer that has progressed following treatment with a fluoropyrimidine, oxaliplatin, and irinotecan	28.0%	TAS-102	1.6%	Mayer et al. [15]
13	Pembrolizumab (Keytruda)	September 22, 2017	Recurrent locally advanced or metastatic, gastric or gastroesophageal junction adenocarcinoma whose tumors express PD-L1. Patients must have had disease progression on or after two or more prior systemic therapies, including fluoropyrimidine- and platinum-containing chemotherapy and, if appropriate, HER2/neu-targeted therapy	13.3%	NA		
14	Nivolumab (Opdivo)	September 22, 2017	HCC in patients who have been previously treated with sorafenib	14.3%	Regorafenib	11%	Bruix et al. [16]
15	Abemaciclib (Verzenio)	September 28, 2017	Monotherapy for women and men with HR-positive, HER2-negative advanced or metastatic breast cancer with disease progression following endocrine therapy and prior chemotherapy in the metastatic setting	19.7%	Eribulin	11.0%	Drugs@FDA [17]
16	Afatinib (Gilotrif)	January 12, 2018	Broadened indication in first-line treatment of patients with metastatic NSCLC whose tumors have non-resistant EGFR mutations	66.0%	Afatinib	50.4%	FDA Drug Approvals and Databases [18]
17	Dabrafenib and Trametinib (Tafinlar and Mekinist)	May 4, 2018	Locally advanced or metastatic anaplastic thyroid cancer with BRAF V600E mutation and with no satisfactory locoregional treatment options	61.0%	Paclitaxel + Carboplatin	16%	Sosa et al. [19]
18	Pembrolizumab (Keytruda)	June 1, 2018	Recurrent or metastatic cervical cancer with disease progression on or after chemotherapy whose tumors express PD-L1 (CPS ≥ 1)	14.3%	Nab-paclitaxel	28.6%	Alberts et al. [20]
19	Ipilimumab (Yervoy)	July 10, 2018	Combination with nivolumab, MSI-H or dMMR metastatic colorectal cancer that has progressed following treatment with a fluoropyrimidine, oxaliplatin, and irinotecan	46.0%	Nivolumab	28%	See the result of #12
20	Nivolumab (Opdivo)	August 16, 2018	Metastatic SCLC with progression after platinum-based chemotherapy and at least one other line of therapy	12.0%	NA		
21	Cemiplimab-rwlc (Libtayo)	September 28, 2018	Metastatic CSCC or locally advanced CSCC who are not candidates for curative surgery or curative radiation	47.0%	Panitumumab	31%	Drugs@FDA [21]
22	Lorlatinib (Lorbrena)	November 2, 2018	ALK-positive metastatic NSCLC whose disease has progressed on crizotinib and at least one other ALK inhibitor for metastatic disease or whose disease has progressed on alectinib or ceritinib as the first ALK inhibitor therapy for metastatic disease	48.0%	Atezolizumab	14%	Drugs@FDA [22]
23	Pembrolizumab (Keytruda)	November 9, 2018	HCC who have been previously treated with sorafenib	17.0%	Nivolumab	14.3%	See the result of #14
24	Pembrolizumab (Keytruda)	December 19, 2018	Recurrent locally advanced or metastatic MCC	56.0%	Avelumab	33.0%	See the result of #6
25	Erdafitinib (Balversa)	April 12, 2019	Locally advanced or metastatic UC, that has: • susceptible FGFR3 or FGFR2 genetic alterations, and • progressed during or following at least one line of prior platinum-containing chemotherapy, including within 12 months of neoadjuvant or adjuvant platinum-containing chemotherapy	32.2%	Pembrolizumab	21.0%	Drugs@FDA [23]
26	Pembrolizumab (Keytruda)	June 17, 2019	Metastatic SCLC with disease progression on or after platinum-based chemotherapy and at least one other prior line of therapy	19.0%	Nivolumab	12.0%	See the result of #20
27	Entrectinib (Rozlytrek)	August 15, 2019	Metastatic NSCLC whose tumors are ROS1-positive	78.0%	Crizotinib	66.0%	Drugs@FDA [24]
28	Pembrolizumab plus Lenvatinib (Keytruda plus Lenvima)	September 17, 2019	Advanced endometrial carcinoma that is not MSI-H or dMMR and who have disease progression following prior systemic therapy but are not candidates for curative surgery or radiation	38.3%	Bevacizumab	13.5%	Aghajanian et al. [25]
29	Niraparib (Zejula)	October 23, 2019	Advanced ovarian, fallopian tube, or primary peritoneal cancer treated with three or more prior chemotherapy regimens and whose cancer is associated with HDR-positive status	24.0%	Olaparib	34.0%	Kim et al. [26]
30	Enfortumab vedotin-ejfv (Padcev)	December 18, 2019	Adult patients with locally advanced or metastatic UC who have previously received a PD-1 or PD-L1 inhibitor, and a platinum-containing chemotherapy in the neoadjuvant/adjuvant, locally advanced or metastatic setting	44.0%	Docetaxel	10.5%	Drakaki et al. [27]
31	Fam-trastuzumab deruxtecan-nxki (Enhertu)	December 20, 2019	Unresectable or metastatic HER2-positive breast cancer who have received two or more prior anti-HER2-based regimens in the metastatic setting	60.3%	T-DM1	31.0%	Krop et al. [28]

*ALK* anaplastic lymphoma kinase, *BRAF* v-RAF murine sarcoma viral oncogene homolog B1, *BRCA* breast cancer susceptibility gene, *CPS* combined positive score, *CSCC* cutaneous squamous cell carcinoma, *dMMR* mismatch-repair deficient, *EGFR* epidermal growth factor receptor, *FGFR* fibroblast growth factor receptor, *HCC* Hepatocellular carcinoma, *HDR* homologous recombination deficiency, *HER2* human epidermal growth factor receptor, *HR* hormone receptor, *MCC* merkel cell carcinoma, *MSI-H* microsatellite instability-high, *NA* not applicable, *NSCLC* non-small cell lung cancer, *PD-1* programmed cell death receptor-1, *PD-L1* programmed cell death ligand 1, *ROS1* c-ros oncogene 1, *SCLC* small cell lung cancer, *UC* urothelial carcinoma

### Comparison of ORRs between the investigated product and BCT

**Fig. 2:**
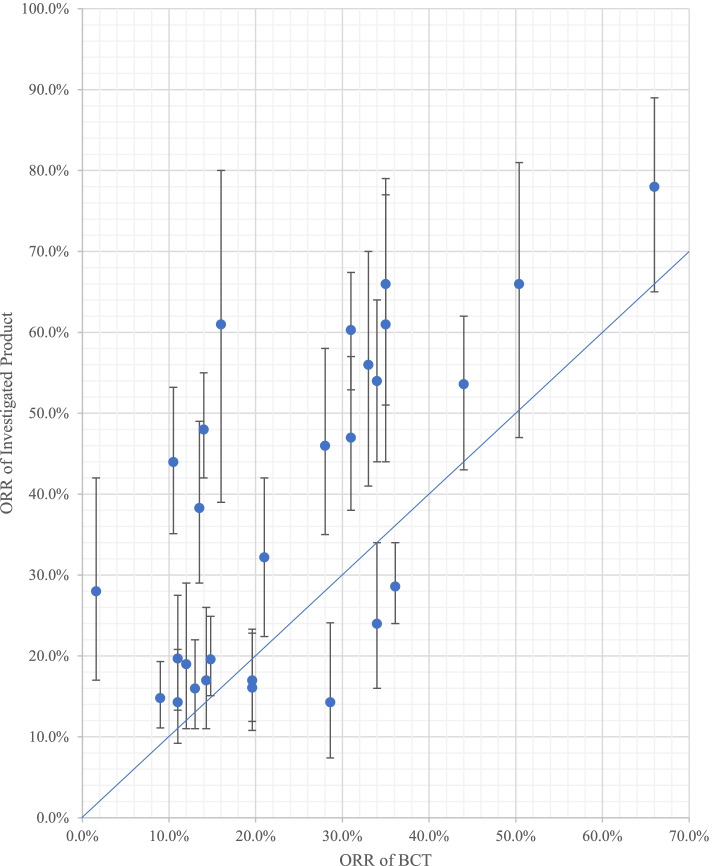
Comparison of ORR between the investigated product and BCT. Scatter plot comparing ORR of investigated product and BCT. The vertical line shows the 95% CI of the ORR of investigated product. *BCT* best comparison therapy, *CI* confidence interval, *ORR* overall response rate

In 23/28 indications (82.1%), the ORR of the investigated product exceeded that of the BCT, and in 16 of these (69.6%), the lower limit of the 95% CI of the ORR of the investigated product exceeded the point estimate of the ORR of the BCT. For seven of these products (7/23), the lower limit of the 95% CI was below the point estimate of the ORR of the BCT, with differences ranging from 1.0% to 3.4% (Fig. 2). For five indications (5/28), the point estimate of the ORR of the investigated product was below that of the BCT: three immune checkpoint inhibitors, i.e., durvalumab (#8), avelumab (#9), and pembrolizumab (#10), for urothelial carcinoma, pembrolizumab (#18) for cervical cancer, and niraparib (#29) for ovarian cancer.

## Discussion

In the present study, the BCTs for each of the indications with SAT approval were identified using objective criteria, and the ORR of the investigated product was compared to that of the BCT. Our results suggested that a 95% CI lower limit of a SAT-based ORR of a new drug that exceeds the point estimate of the ORR of the BCT could be an important factor in deciding on approval of the new drug.

It is well-recognized that a high SAT-based ORR is required for new drug approval. In the European Society for Medical Oncology Magnitude of Clinical Benefit Scale (ESMO-MCBS) V1.1, Evaluation Form 3 [29] provides three grades for evaluation of SATs when the primary endpoint is the ORR or progression-free survival. The ORR grade is classified by the degree of the ORR alone or its combination with the duration of response (DOR). For example, an ORR > 60% is rated as Grade 3, while an ORR of 40‒60% is considered as Grade 2. Thus, a high ORR is highly valued. In this study, the ORRs of the 28 investigated products ranged from 14.3% to 78.0%. For 13 products (46.4%), the ORRs exceeded 40%. Of these, 11 products were molecular targeted drugs or antibody‒drug conjugates. Their high anti-tumor efficacy was demonstrated based on their MOA, which led to their approval. Ten of the 28 products (35.7%) had ORRs of 10‒20% (Grade 1 by ESMO-MCBS criteria). Nine of these products were anti-programmed cell death 1 (PD-1)/programmed cell death ligand 1 (PD-L1) antibodies, which show long-term responses [30]. These products likely obtained approval based on their efficacy, including the DOR, despite their low ORRs. Nevertheless, regardless of the magnitude of the ORR, the lower limit of the 95% CI of the ORR of the investigated product tended to exceed the point estimate of the BCT ORR, suggesting that this could be an important factor in approving the new drug.

There were five indications approved with an ORR below the point estimate of the BCT ORR. The review report for durvalumab, which was approved for second-line urothelial carcinoma, stated that vinflunine was evaluated as a historical control. At the same time, avelumab was also approved for the same indication. The lower limit of the 95% CI of the ORRs of both products exceeded the ORR of 9% for vinflunine. The review report for durvalumab also stated that the ORR was similar to that of other immune checkpoint inhibitors, which had been identified as a BCT in the present study, and it was superior to that of the available chemotherapy. For SAT-based approval, it would be important to establish a comparator that is acceptable to the FDA and that the lower limit of the 95% CI of the new drug’s ORR exceeds the ORR of a comparator, rather than comparing it to the latest available therapy at the time of approval.

For pembrolizumab as first-line urothelial cancer, the ORR was 32.3% (95% CI: 26.8‒38.1) in a subgroup analysis of patients with PD-L1 combined positive scores (CPSs) ≥ 1%, and 47.3% (95% CI: 37.7. 57.0) in those with a CPS ≥ 10% [31]. For the patient population with a CPS ≥ 10%, the lower limit of the 95% CI for pembrolizumab exceeded the point estimate of the BCT (gemcitabine plus carboplatin) ORR. The NCCN guidelines [32] recommend pembrolizumab for patients with a CPS ≥ 10%, although it is indicated for cisplatin-ineligible urothelial cancer cases, regardless of PD-L1 expression. For pembrolizumab as second-line treatment for cervical cancer, the NCCN guidelines [33] recommend it for patients with a PD-L1 CPS > 1 and DNA mismatch-repair deficient (dMMR) or microsatellite instability-high (MSI-H) cases, but for all other patient subpopulations, recommendations for this drug by the guidelines are rated as category 2B. Pembrolizumab was likely approved as a drug with an expected long DOR, although the ORR was inferior to chemotherapy, in situations with little consensus data.

For niraparib for late-line ovarian cancer, olaparib was expected to be used for patients with *BRCA* mutations, and niraparib for homologous recombination deficiency (HRD)-positive patients. Niraparib was thought to be approved because some study results showed efficacy in clear patient populations and late-line treatment options are limited.

Ladanie et al. reported that 87% of anticancer drugs approved with SAT results and 50% of anticancer drugs approved with RCT results had received orphan drug designation during 2000‒2016 [3]. SATs are considered to be a drug development strategy mainly adopted for new drug applications for rare cancers, in which it is difficult to conduct confirmatory studies. Yet, 28.6% (10/35) of products with SAT-based approval, and 21.2% (14/66) of products with RCT-based approval had received orphan drug designation in the present study, for data collected during 2016‒2019. This suggests that the drug development strategy utilizing SATs as pivotal trials is no longer limited to rare cancers. Additionally, even drugs that do not necessarily have a high ORR, such as the newer anti-PD-1/PD-L1 inhibitors, may be considered to have a suitably high ORR, if the sample size were such that the ORR would slightly but statistically significantly exceed the ORR of available therapies. This suggests that the environment for development strategies based on SATs has changed, which may have enhanced SAT-based approvals. On the other hand, Gyawali et al. reported on some anticancer drugs that received AA but failed to improve the primary endpoint in post-approval confirmatory trials [34]. It indicated the importance of understanding the difficulty of evaluating the clinical benefit of new treatments based on limited information such as ORR.

This study had some limitations. In this study, only approved drugs were included in the analysis, and unapproved or unfiled drugs were not investigated. There might have been some drugs that showed sufficient ORR in the SAT, but were not approved for some reason; however, it was difficult to identify these facts from the published information. This is an issue for future research.

## Conclusions

Our results suggested that a lower 95% CI limit for the new drug ORR in an SAT that exceeds the point estimate of the BCT ORR, could be an important factor in obtaining regulatory approval. Thus, the expected value of the ORR should be set according to the MOA of the new drug, by referencing the ORR of an available therapy as a benchmark.

## Data Availability

The datasets used and/or analysed during the current study are available from the corresponding author on reasonable request.

## Abbreviations

SAT: Single-arm trial
ORR: Objective response rate
FDA: Food and Drug Administration
BCT: Best comparison therapy
CI: Confidence interval
RCTs: Randomized controlled trials
OS: Overall survival
RECIST: Response Evaluation Criteria in Solid Tumors
CT: Computed tomography
AA: Accelerated Approval
MOA: Mechanism of action
NCCN: National Comprehensive Cancer Network
ESMO-MCBS: European Society for Medical Oncology Magnitude of Clinical Benefit Scale
DOR: Duration of response
PD-1: Programmed cell death 1
PD-L1: Programmed cell death ligand 1
CPSs: Combined positive scores
dMMR: DNA mismatch-repair deficient
MSI-H: Microsatellite instability-high
HRD: Homologous recombination deficiency

## References

[1] European Medicines Agency (EMA). Guideline on the evaluation of anticancer medicinal products in man. eu/en/documents/scientific -guide line/guideline-evaluation -anticancer -medic inal-products-man-revision-5_en.pdf. https://www.ema.europa. Accessed 7 Feb 2021.

[2] Clinical trial endpoints for the approval of cancer drugs and biologic guidance for industry. https://www.fda.gov/regulatory-information/search-fda-guidance-documents/clinical-trial-endpoints-approval-cancer-drugs-and-biologics. Accessed 7 Feb 2021.

[3] Ladanie A, Speich B, Briel M, Sclafani F, Bucher HC, Agarwal A (2019). Single pivotal trials with few corroborating characteristics were used for FDA approval of cancer therapies. J Clin Epidemiol.

[4] Eisenhauera EA, Therasseb P, Bogaertsc J, Schwartz LH, Sargent D, Ford R (2009). New response evaluation criteria in solid tumours: Revised RECIST guideline (version 1.1).. Eur J Cancer..

[5] Guidance for industry expedited programs for serious conditions drugs and biologics. https://www.fda.gov/regulatory-information/search-fda-guidance-documents/expedited-programs-serious-conditions-drugs-and-biologics. Accessed 7 Feb 2021.

[6] Oxnard GR, Wilcox KH, Gonen M, Polotsky M, Hirsch BR, Schwartz LH (2016). Response rate as a regulatory end point in single-arm studies of advanced solid tumors. JAMA Oncol.

[7] Hematology. Oncology (Cancer) Approvals & Safety Notifications. https://www.fda.gov/drugs/informationondrugs/approveddrugs/ucm279174.htm. Accessed 20 Jan 2020.

[8] Drugs@FDA. https://www.accessdata.fda.gov/scripts/cder/daf/index.cfm.

[9] Sandler A, Gray R, Perry MC, Brahmer J, Schiller JH, Dowlati A (2006). Paclitaxel–carboplatin alone or with bevacizumab for non-small-cell lung cancer. N Engl J Med.

[10] Drugs@FDA. https://www.accessdata.fda.gov/drugsatfda_docs/nda/2016/761034Orig1s000TOC.cfm. Accessed 14 Feb 2021.

[11] Vermorken JB, Trigo J, Hitt R, Koralewski P, Diaz-Rubio E, Rolland F (2007). Open-label, uncontrolled, multicenter phase II study to evaluate the efficacy and toxicity of cetuximab as a single agent in patients with recurrent and/or metastatic squamous cell carcinoma of the head and neck who failed to respond to platinum-based therapy. J Clin Oncol.

[12] Drugs@FDA. https://www.accessdata.fda.gov/drugsatfda_docs/nda/2016/209115Orig1s000TOC.cfm. Accessed 14 Feb 2021.

[13] Drugs@FDA. https://www.accessdata.fda.gov/drugsatfda_docs/nda/2017/208772Orig1s000TOC.cfm. Accessed 14 Feb 2021.

[14] Santis MD, Bellmunt J, Mead G, Kerst JM, Leahy M, Maroto P (2012). Randomized phase II/III trial assessing gemcitabine/carboplatin and methotrexate/carboplatin/vinblastine in patients with advanced urothelial cancer who are unfit for cisplatin-based chemotherapy: EORTC Study 30986. J Clin Oncol.

[15] Mayer RJ, Van Cutsem E, Falcone A, Yoshino T, Garcia-Carbonero R, Mizunuma N (2015). Randomized trial of TAS-102 for refractory metastatic colorectal cancer. N Engl J Med.

[16] Bruix J, Qin S, Merle P, Granito A, Huang YH, Bodoky G (2017). Regorafenib for patients with hepatocellular carcinoma who progressed on sorafenib treatment (RESORCE): A randomised, double-blind, placebo-controlled, phase 3 trial. Lancet.

[17] Drugs@FDA. https://www.accessdata.fda.gov/drugsatfda_docs/nda/2017/208716Orig1s000TOC.cfm. Accessed 14 Feb 2021.

[18] FDA drug approvals and databases. http://wayback.archive-it.org/7993/20170111231713/http:/www.fda.gov/Drugs/InformationOnDrugs/ApprovedDrugs/ucm360574.htm. Accessed 14 Feb 2021.

[19] Sosa JA, Elisei R, Jarzab B, Balkissoon J, Lu SP, Bal C (2014). Randomized safety and efficacy study of fosbretabulin with paclitaxel/carboplatin against anaplastic thyroid carcinoma. Thyroid.

[20] Alberts DS, Blessing JA, Landrum LM, Warshal DP, Martin LP, Rose SL (2012). Phase II trial of nab-paclitaxel in the treatment of recurrent or persistent advanced cervix cancer: A gynecologic oncology group study. Gynecol Oncol.

[21] Drugs@FDA. https://www.accessdata.fda.gov/drugsatfda_docs/nda/2018/761097Orig1s000TOC.cfm. Accessed 14 Feb 2021.

[22] Drugs@FDA. https://www.accessdata.fda.gov/drugsatfda_docs/nda/2018/210868Orig1s000TOC.cfm. Accessed 14 Feb 2021.

[23] Drugs@FDA. https://www.accessdata.fda.gov/drugsatfda_docs/nda/2019/212018Orig1s000TOC.cfm. Accessed 14 Feb 2021.

[24] Drugs@FDA. https://www.accessdata.fda.gov/drugsatfda_docs/nda/2019/212725Orig1s000,%20212726Orig1s000TOC.cfm. Accessed 14 Feb 2021.

[25] Aghajanian C, Sill MW, Darcy KM, Greer B, McMeekin DS, Rose PG (2011). Phase II trial of bevacizumab in recurrent or persistent endometrial cancer: A Gynecologic Oncology Group study. J Clin Oncol.

[26] Kim G, Ison G, McKee AE, Zhang H, Tang S, Gwise T (2015). FDA approval summary: Olaparib monotherapy in patients with deleterious germline BRCA-mutated advanced ovarian cancer treated with three or more lines of chemotherapy. Clin Cancer Res.

[27] Drakaki A, Kirby CJ, Van der Heijden MS, Petrylak DP, Powles T, Chi KN (2018). Docetaxel with or without ramucirumab after immune checkpoint inhibition in platinum-refractory metastatic urothelial carcinoma (mUC): Prespecified subgroup analysis from the phase 3 RANGE trial. J Clin Oncol..

[28] Krop IE, Kim S-B, González-Martín A, LoRusso PM, Ferrero J, Smitt M (2014). Trastuzumab emtansine versus treatment of physician’s choice for pretreated HER2-positive advanced breast cancer (TH3RESA): A randomised, open-label, phase 3 trial. Lancet Oncol.

[29] ESMO-MCBS Version-1.1 Evaluation Form 3. https://www.esmo.org/content/download/117393/2059180/1/ESMO-MCBS-Version-1-1-Evaluation-Form-3.pdf. Accessed 7 Feb 2021.

[30] Gauci ML, Lanoy E, Champiat S, Caramella C, Ammari S, Aspeslagh S (2019). Long-term survival in patients responding to anti-PD-1/PD-L1 therapy and disease outcome upon treatment discontinuation. Clin Cancer Res.

[31] Study of pembrolizumab (MK-3475) in participants With advanced urothelial cancer (MK-3475-052/KEYNOTE-052) - Study results. *Clin Trials*.gov. https://clinicaltrials.gov/ct2/show/results/NCT02335424. Accessed 7 Feb 2021.

[32] National Comprehensive Cancer Network (NCCN) (2020). NCCN clinical practice guidelines in oncology (NCCN guidelines) for bladder cancer.

[33] NCCN. NCCN guidelines for cervical cancer 2020. https://www.nccn.org/professionals/physician_gls/pdf/cervical.pdf. Accessed 7 Feb 2021.

[34] Gyawali B, Rome BN, Kesselheim AS (2021). Regulatory and clinical consequences of negative confirmatory trials of accelerated approval cancer drugs: retrospective observational study. BMJ.

